# Mitochondrial Tethers and Their Impact on Lifespan in Budding Yeast

**DOI:** 10.3389/fcell.2017.00120

**Published:** 2018-01-08

**Authors:** Wolfgang M. Pernice, Theresa C. Swayne, Istvan R. Boldogh, Liza A. Pon

**Affiliations:** ^1^Department of Pathology and Cell Biology, Columbia University, New York, NY, United States; ^2^Herbert Irving Comprehensive Cancer Center, Columbia University, New York, NY, United States

**Keywords:** mitochondria, lifespan, budding yeast, asymmetric cell division, organelle contact sites

## Abstract

Tethers that link mitochondria to other organelles are critical for lipid and calcium transport as well as mitochondrial genome replication and fission of the organelle. Here, we review recent advances in the characterization of interorganellar mitochondrial tethers in the budding yeast, *Saccharomyces cerevisiae*. We specifically focus on evidence for a role for mitochondrial tethers that anchor mitochondria to specific regions within yeast cells. These tethering events contribute to two processes that are critical for normal replicative lifespan: inheritance of fitter mitochondria by daughter cells, and retention of a small pool of higher-functioning mitochondria in mother cells. Since asymmetric inheritance of mitochondria also occurs in human mammary stem-like cells, it is possible that mechanisms underlying mitochondrial segregation in yeast also operate in other cell types.

## Introduction

Mitochondria have emerged as central regulators of lifespan through multiple mechanisms. Mitochondria are the site for generation of intermediary metabolites including acetyl-CoA and NAD^+^/NADH, which regulate histone deactylases including the sirtuin family of age modulators (Starai et al., 2002; Hallows et al., 2006). Mitochondria are also the site for biosynthesis of iron-sulfur clusters (Braymer and Lill, 2017). Defects in this process result in nuclear genome instability, one of the hallmarks of aging (Veatch et al., 2009). Moreover, mitochondria serve as signaling platforms that affect lifespan by activation of stress response and quality control pathways. For example, in *C. elegans* and *Drosophila*, mild mitochondrial stress induced by mutation of respiratory chain components results in lifespan extension. These effects are a consequence of activation of the mitochondrial unfolded protein response (UPR^mt^), a pathway that up-regulates mitochondrial proteostasis, antioxidant defenses and mitochondrial biosynthesis (Durieux et al., 2011; Owusu-Ansah et al., 2013). Mitochondrial stress can also lead to healthspan extension by delaying the age-linked decline in the heat shock response (HSR) pathway that maintains protein quality control within the cytosol and nucleus (Labbadia et al., 2017). Finally, selective autophagy of damaged mitochondria, a process that is driven by ubiquitination of mitochondrial proteins by the E3 ubiquitin ligase Parkin, illustrates the importance of mitochondrial quality control in health- and lifespan. Deletion or overexpression of Parkin shortens (Greene et al., 2003) or extends lifespan, respectively, in *Drosophila* (Rana et al., 2013). In addition, mutation of Parkin or Pink1, a protein that recruits Parkin to mitochondria, is associated with familial Parkinson's disease, an age-associated neurodegenerative disease (Kitada et al., 1998; Valente et al., 2004).

A fundamentally different mechanism whereby mitochondria impact lifespan has been identified in cells undergoing asymmetric cell division, the process in which differential segregation of cellular constituents generates daughter cells that are not identical. In higher eukaryotes, including humans, asymmetric division is critical for stem cell function. It allows for the simultaneous renewal of stem-cell properties in one daughter cell and the production of a second, differentiating daughter cell that regenerates specific organs and tissues as they age (Ouellet and Barral, 2012). Indeed, it is possible that defects in asymmetric stem cell division contribute to the age-associated declines in stem cell number and function in regeneration of hematopoietic cells (Shaw et al., 2010), mouse forebrain (Molofsky et al., 2006), bone (Gruber et al., 2006), and skeletal muscle (Conboy and Rando, 2012). The budding yeast, *Saccharomyces cerevisiae*, also undergoes asymmetric cell division. One consequence of this is mother-daughter age asymmetry. Yeast mother cells have a finite replicative lifespan: they can produce an average of ~30 buds. While yeast mother cells continue to age as they replicate, daughter cells are born young, with their full replicative lifespan (Jazwinski, 1990; Kennedy et al., 1994).

Here, we outline the effect of the non-uniform segregation of mitochondria during asymmetric cell division on cell fate and lifespan. In particular, we focus on the role of interorganellar mitochondrial contact sites in several key homeostatic processes in yeast and metazoans and in control of mitochondrial distribution during asymmetric inheritance of the organelle.

## Segregation of mitochondria during asymmetric cell division

### Asymmetric inheritance of mitochondria in human mammary stem-like cells

**Figure 1:**
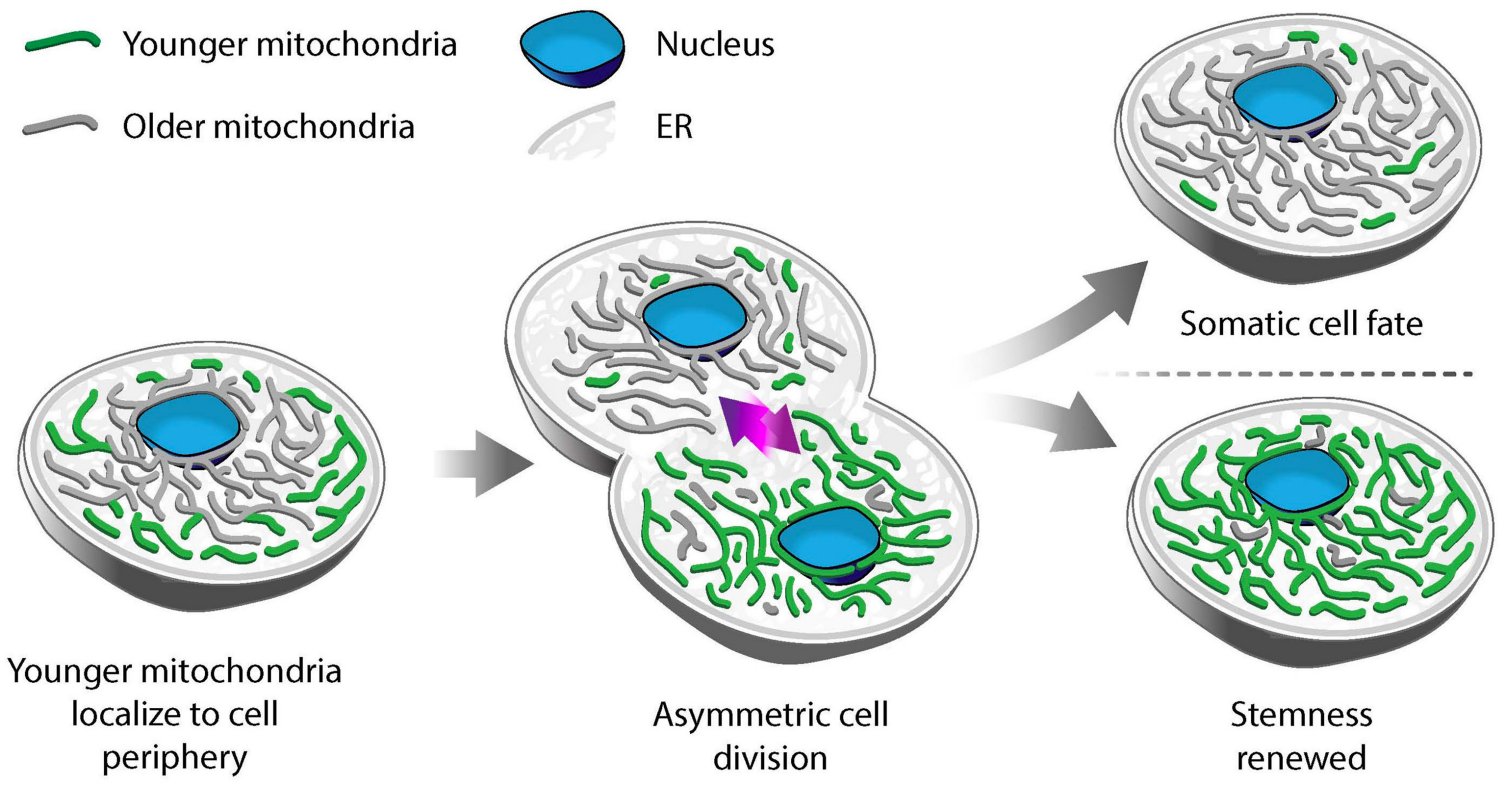
Asymmetric segregation of mitochondria in stem-like cells. In mammary epithelial stem-like cells, a model for asymmetric stem cell division, younger mitochondria (green) localize to the cell periphery, while older mitochondria (gray) are perinuclear. Upon cell division, younger mitochondria are preferentially segregated to the daughter cell that undergoes self-renewal and retains stem-like properties. Older mitochondria are inherited by the daughter cell that differentiates to a tissue progenitor cell.

Using photoconvertible fluorescent labels to differentially label newly generated and older mitochondria, Katajisto and colleagues identified asymmetric inheritance of mitochondria in human mammary stem-cell-like cells (Katajisto et al., 2015). They found that daughter cells that maintained more stem-like characteristics preferentially inherited newer mitochondria, while daughter cells destined to differentiate and develop epithelial characteristics preferentially inherited older mitochondria (Figure 1). Interestingly, maintaining stem cell properties correlates not just with the age of mitochondria but also with mitochondrial function: cells that contain mitochondria with higher membrane potential (ΔΨ) show increased mammosphere-forming capacity, which is a measure of stem cell function. Stem-like cells also exhibit increased mitophagy, and therefore increased capacity to remove damaged mitochondria, compared to daughter cells destined to differentiate. Consistent with this, inhibition of *Parkin*, a protein that marks mitochondria for mitophagy, reduces asymmetric inheritance of the organelle.

Differential localization is another mechanism for segregation of mitochondria by function in human mammary stem-like cells. Old mitochondria are more likely to localize near the nucleus, while younger ones are distributed more evenly throughout the cytoplasm in asymmetrically dividing stem-like cells. Treatment with an inhibitor of mitochondrial division (mdivi-1), or overexpression of the fission-inducing protein *Drp1*, results in mislocalization of old mitochondria throughout the cytoplasm, decreased segregation of old from young mitochondria and loss of stem cell properties in daughter cells (Katajisto et al., 2015). While it is clear that the asymmetric inheritance of mitochondria in human mammary stem-like cells is affected by the dynamics and localization of the organelle, the mechanism underlying this process is not well understood.

### Asymmetric mitochondrial inheritance in budding yeast

During asymmetric cell division in budding yeast, mitochondria are actively partitioned between the mother and the developing bud, and accumulate at opposite cellular poles: the bud tip and the distal tip of the mother cell. The poleward displacements are achieved by both anterograde movements (toward the bud) and retrograde movements (toward the distal tip of the mother) (Fehrenbacher et al., 2004).

**Figure 2:**
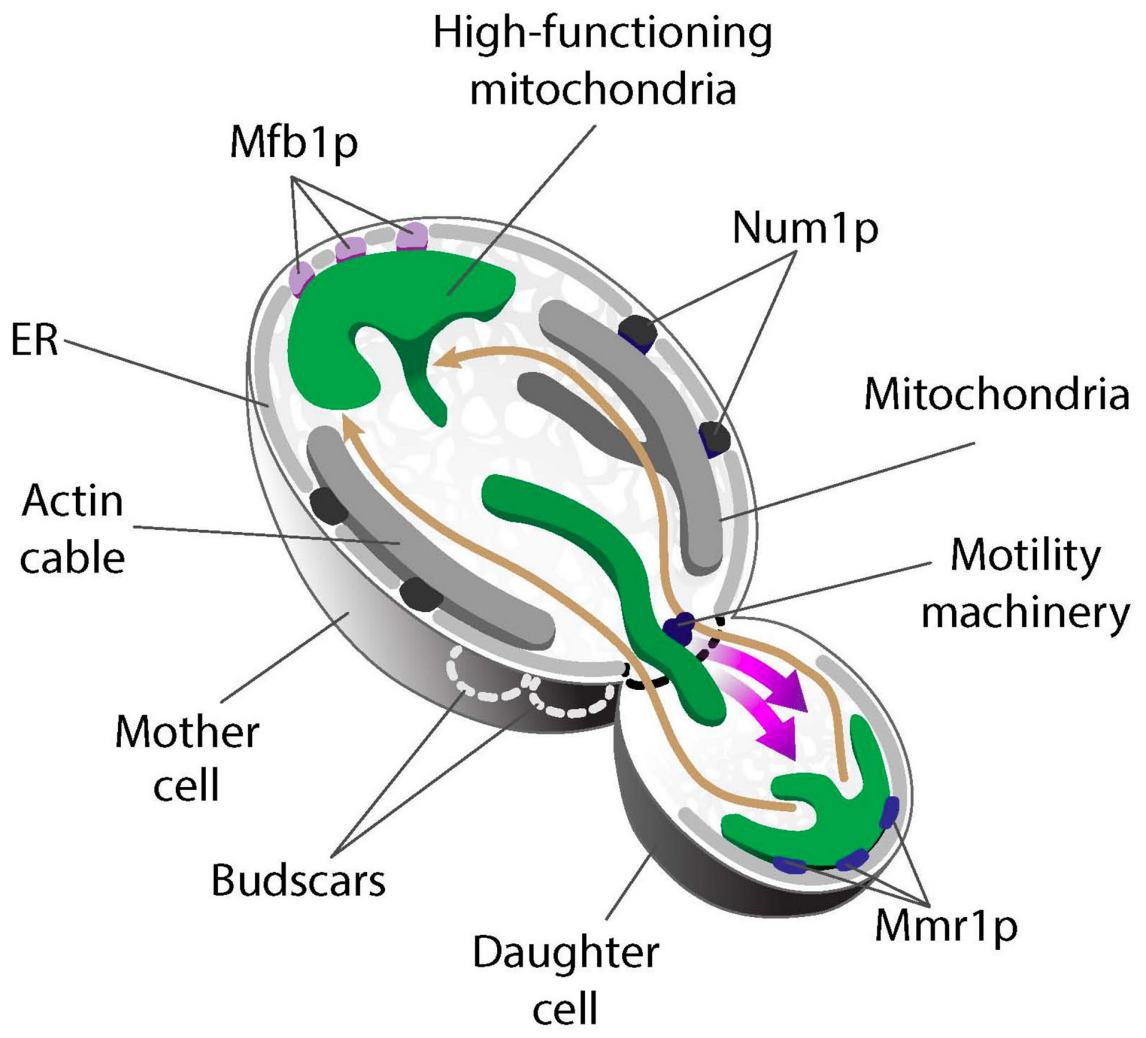
Asymmetric mitochondrial inheritance in yeast. Higher-functioning mitochondria (green) localize to both the bud tip and tip of the mother cell in *S. cerevisiae*. This is achieved by the coordinated effort of the mitochondrial motility machinery and region-specific anchorage of high-functioning mitochondria during cell division. Mitochondria are transported from mother cells to buds along actin cables, dynamic tracks that exhibit retrograde flow (movement from buds to mother cells). High-functioning mitochondria are more motile and therefore able to overcome the opposing force of retrograde actin cable flow and move into buds. These higher-functioning mitochondria are anchored to cER in the bud tip by Mmr1. Two tethers retain mitochondria in mother cells: Num1 mediates mitochondrial tethering throughout the maternal cortex, but does not contribute to mitochondrial quality control during inheritance. In contrast, Mfb1 localizes to and mediates anchorage and retention of high-functioning mitochondria specifically at the mother cell tip. Loss of function of either Mmr1 or Mfb1 results in defects in mitochondrial quality control and altered lifespan.

Recent studies exploring mitochondrial quality parameters as a function of subcellular localization found that yeast daughter cells contain mitochondria with less reactive oxygen species (ROS), higher reducing potential and higher ΔΨ compared to mitochondria in mother cells (McFaline-Figueroa et al., 2011; Higuchi et al., 2013; Pernice et al., 2016). Thus, fitter mitochondria are preferentially inherited by yeast daughter cells. In contrast, there are some high- and low-functioning mitochondria in mother cells. As a result, the overall function of mitochondria in mother cells is lower than that of mitochondria in buds. Interestingly, the small population of higher-functioning mitochondria that are present in mother cells localize to the tip of the mother cell that is distal to the bud (mother cell tip) (Figure 2) (Pernice et al., 2016).

Live-cell imaging revealed a role for mitochondrial motility, dynamics, and region-specific tethering of the organelle in the asymmetric inheritance of mitochondria in budding yeast (Figure 2). First, actin cables, the tracks for mitochondrial movement, are dynamic structures that move from the bud toward the mother cell tip (Yang and Pon, 2002; Huckaba et al., 2006). As a result, fitter mitochondria that exhibit higher anterograde motility rates and can overcome the opposing force of retrograde actin cable flow are preferentially transported from mother to daughter cell (Higuchi et al., 2013). Second, fluorescence loss in photobleaching studies revealed that mitochondria in the bud can be functionally distinct from mitochondria in mother cells, in part because they are physically distinct (McFaline-Figueroa et al., 2011). Third, higher-functioning mitochondria are retained at the opposite poles of the yeast cell because they are anchored and immobilized at those sites. Mitochondria at the bud tip are tethered to the cortical endoplasmic reticulum (cER), an ER network that localizes to the cell periphery and is itself anchored to the plasma membrane (PM) (Swayne et al., 2011). Interestingly, deletion of the 5 genes that mediate cER-PM interactions (Manford et al., 2012) has no effect on accumulation of high-functioning mitochondria in the mother cell tip (Pernice et al., 2016). Thus, distinct mechanisms mediate anchorage of high-functioning mitochondria at the opposite poles of the budding yeast cell. Below, we describe the role for tethers in asymmetric inheritance of mitochondria and in lifespan control.

## Tethers that link mitochondria to other organelles

Organelles were once believed to be physically and functionally distinct subcellular compartments. However, it is now clear that mitochondria interact with other organelles including the ER, PM, vacuoles (the yeast lysosome), and peroxisomes (Murley and Nunnari, 2016). Several of these contact sites appear to function primarily in controlling mitochondrial distribution. Other interorganellar mitochondrial contact sites function in key biosynthetic and signaling pathways.

### Mitochondrial interorganellar contact sites that function in cell metabolism and signaling

One primary function of mitochondrial contact sites with other organelles is the exchange of lipids between the apposed membranes. Mitochondria must import phospholipids, including phosphatidylcholine (PC), and precursors for phosphatidylethanolamine (PE) and cardiolipin (CL) biosynthesis from the ER (Vance, 2015). The ER-mitochondrial encounter structure (ERMES) of *S. cerevisiae* (Kornmann et al., 2009) was the first tether identified that links mitochondria to ER. It consists of mitochondrial outer membrane proteins and an integral ER membrane protein. Three out of four ERMES subunits contain a synaptotagmin-like mitochondrial-lipid binding protein (SMP) domain (Lee and Hong, 2006; Kopec et al., 2010), suggesting that ERMES proteins facilitate lipid transport directly (Schauder et al., 2014). Surprisingly, deletion of ERMES subunits has only subtle effects on the levels of aminoglycerophospholipids in mitochondria, phospholipids that are produced at sites of ER-mitochondrial contact (Kornmann et al., 2009; Nguyen et al., 2012; Voss et al., 2012).

Further analysis of ERMES mutants led to the identification of another mechanism for lipid transport at mitochondria: contact sites between mitochondria and the vacuole (vCLAMPs). Schuldiner and colleagues found that deletion of 2 proteins previously implicated in vacuolar fusion (Vps39 and Vam7) (Price et al., 2000; Stroupe et al., 2006) results in an increase in ERMES (Elbaz-Alon et al., 2014). They also found that Vps39 localizes to vCLAMPs, vCLAMPs expand in ERMES mutants, and repression of ERMES subunits in *vps39*Δ cells results in defects in phospholipid composition of mitochondria (Elbaz-Alon et al., 2014). In complementary studies, Honscher et al. found that deletion of Vps39 results in a decrease in vCLAMPs and that overexpression of Vps39 increases the size of vCLAMPS and rescues growth defects observed in ERMES mutants (Hönscher et al., 2014). Finally, recent studies revealed that Lam6, a conserved protein that contains GRAM lipid-binding domains (Doerks et al., 2000; Gatta et al., 2015), co-immunoprecipitates with multiple ERMES subunits and co-localizes with ERMES (Elbaz-Alon et al., 2015). Interestingly, Lam6 also localizes to vCLAMPs and to nucleus-vacuolar junctions (NVJs) and overexpression of *LAM6* results in expansion of all 3 junctions (Elbaz-Alon et al., 2015). Overall, these studies indicate that vCLAMPs and ERMES have redundant functions in lipid transport to mitochondria and that Lam6 plays a role in regulating the cross-talk between multiple organelle contact sites. Yet to be determined is the mechanism underlying vCLAMP function in lipid transport to mitochondria.

ER-mitochondria contact sites also participate in processes other than lipid transport. For example, they function in calcium ion (Ca^2+^) transport between the organelles (Rizzuto et al., 1998; Stone and Vance, 2000). Recent studies indicate that the SMP domain-containing protein *Pdzd8* localizes to ER at sites of mitochondrial-ER interaction, mediates interactions between mitochondria and ER in mammalian cells, and is required for synaptically induced Ca^2+^ transport between the two organelles (Hirabayashi et al., 2017). Interestingly, mtDNA nucleoids also localize to mitochondria-ER junctions and undergo DNA replication at those sites (Lewis et al., 2016). Finally, these contact sites contribute to mitochondrial fission. Seminal studies revealed that ER tubules wrap around mitochondria and recruit the actin cytoskeleton to that site. Actin then generates contractile forces at the mitochondria-ER interface, leading to assembly of dynamin-related protein 1 (Drp1) at the site of constriction and further contraction of the organelle (Friedman et al., 2011; Korobova et al., 2013). Thus, mitochondria-ER interactions affect fundamental processes, including lipid biogenesis and transport, calcium homeostasis, and mitochondrial dynamics and genome replication, which ultimately affect cellular fitness.

### Mitochondrial tethers that control mitochondrial distribution during asymmetric cell division and affect lifespan

As described above, the mitochondrial motility machinery promotes inheritance of fitter mitochondria by yeast daughter cells, which in turn affects daughter cell fitness and lifespan. Region-specific retention of mitochondria has also emerged as an important mechanism that contributes to the faithful partitioning of the organelle and mitochondrial quality control during yeast cell division. Three major retention mechanisms have been identified in yeast: bud tip-specific tethering by Mmr1; cortical maternal tethering through Num1; and mother tip-specific retention through Mfb1. We here describe how these proteins contribute to mitochondrial quality control and lifespan in yeast.

#### Mmr1: a bud-tip tether that affects quantity and quality of mitochondrial inheritance

Mmr1 was identified in a screen for genetic interactions with the type V myosin motor Myo2 (Itoh et al., 2004). Mmr1 binds to the Myo2 tail and also to unknown factors on mitochondria, suggesting that it might be a receptor for a motor protein that drives mitochondrial movement on actin cables (Eves et al., 2012). However, a more complex picture of Mmr1 function has emerged. *MMR1* protein and mRNA localize to the bud tip (Shepard et al., 2003), which suggests that Mmr1 functions in the bud tip and not in the mother cell where there are high levels of mitochondrial motility. Indeed, Mmr1 localizes to the interface between mitochondria and cER in the bud tip. Moreover, deletion of *MMR1* or failure to localize the protein to the bud tip results in defects in accumulation of mitochondria in the bud tip (Swayne et al., 2011). In addition, cells bearing a temperature-sensitive mutation in *MMR1* release their mitochondria from the bud tip when shifted to restrictive temperature (Higuchi-Sanabria et al., 2016). Thus, Mmr1 appears to be a multifunctional tether that links mitochondria to a myosin for movement, and to cER in the bud tip for inheritance (Figure 2). Interestingly, the localization and deletion phenotype of *MMR1* are fundamentally different from those of ERMES mutants. Therefore, available evidence suggests that Mmr1-mediated anchorage of mitochondria at the bud tip occurs through a mechanism that does not rely on ERMES.

Mmr1 also contributes to control of mitochondrial quality during asymmetric cell division, which in turn affects lifespan. Deletion of *MMR*1 results in the generation of two populations of yeast (McFaline-Figueroa et al., 2011). One population of *mmr1*Δ cells has reduced replicative lifespan and elevated ROS levels. The other population of *mmr1*Δ cells are longer-lived and contain less ROS than wild-type cells. Taken together, these observations led to the model that Mmr1-mediated tethering of higher-functioning mitochondria in the yeast bud tip contributes to retention of higher-functioning mitochondria in the bud, which in turn affects daughter cell fitness and lifespan (Higuchi-Sanabria et al., 2014).

#### Maternal mitochondrial retention in budding yeast

Num1 together with Mdm36 forms the Mitochondria-ER Cortex Anchor (MECA) structure. MECA anchors mitochondria to the PM in yeast mother cells. Specifically, deletion of *NUM1* abolishes cortical anchorage of mitochondria in mother cells and impairs maternal mitochondrial retention, which results in disproportionate inheritance of mitochondria by daughter cells (Klecker et al., 2013; Lackner et al., 2013). Num1 forms punctate structures that distribute throughout the maternal cortex and—as the name implies—both ER and mitochondria are consistently present at Num1 foci. Although MECA does not directly partake in ER-mitochondria tethering (Lackner et al., 2013), it is possible that MECA stabilizes ERMES-mediated tethering of the two organelles. Indeed, Num1 deletion causes severe defects in mitochondrial fission, an event that occurs at mitochondrial-ER contact sites (Cerveny et al., 2007; Klecker et al., 2013).

In contrast to Num1, which controls the *quantity* of mitochondria in yeast mother cells, the mitochondrial F-box-containing protein Mfb1 controls the *quality* of maternal mitochondria (Pernice et al., 2016; Kraft and Lackner, 2017). Mfb1 was originally described as a mitochondrial morphology regulator that is enriched in mother cells (Dürr et al., 2006; Kondo-Okamoto et al., 2006). However, Mfb1 has since been shown to control mitochondrial localization and cell lifespan. Deletion of *MFB1* results in depletion of mitochondria specifically from the mother cell tip. Equally important, there is no accumulation of higher-functioning mitochondria in the mother cell tip in *mfb1*Δ cells. Moreover, overall mitochondrial function is severely compromised in *mfb1*Δ cells (Pernice et al., 2016). Finally, deletion of *MFB1* results in a significant decrease in replicative lifespan. These findings indicate that anchorage of a small population of higher-functioning mitochondria in the mother cell tip is mediated by Mfb1 and that this process affects cellular fitness and lifespan. Since Mfb1 co-localizes with mitochondria that are anchored in the mother cell tip, it is likely that it has a direct role as a tether for mitochondria at that site (Figure 2).

What could be the mechanism for mitochondrial quality control by Mfb1? Treatment of yeast with agents that damage mitochondria, including oxidizing agents or small molecules that reduce ΔΨ, has no effect on Mfb1 localization or function in anchorage of mitochondria in the mother cell tip. Thus, Mfb1 is not actively sensing mitochondrial function (Pernice et al., 2016).

Instead, the association of Mfb1 with a high-functioning mitochondrial population may occur via indirect mechanisms. In particular, this association appears to be linked to patterns of cell polarity. Yeast haploid cells display an axial budding pattern: new buds always form adjacent to the previous bud site. As a result, the bud tip of a daughter cell becomes the mother cell tip in the daughter's next round of cell division (Bi and Park, 2012; Martin and Arkowitz, 2014). Interestingly, during telophase, some Mfb1 localizes to the bud tip where it functions as an anchor for mitochondria. Moreover, Mfb1 that is present at the bud tip remains at that site after cytokinesis, as the cell's polarity reverses and the old bud tip becomes the new mother cell tip. Hence, instead of actively sensing mitochondrial quality, cell cycle-regulated localization of Mfb1 to the bud tip may allow it to capture high-functioning mitochondria at the bud tip and anchor them in the new mother cell tip as the newborn cell begins to replicate (Pernice et al., 2016).

## Conclusion

Overall, these studies indicate that inheritance of fitter mitochondria by daughter cells during asymmetric division in yeast relies on a tether in the bud (Mmr1) that retains high-functioning mitochondria in the bud by anchorage of mitochondria to cER at that site. It also relies on a tether in mother cells (Mfb1) that retains a small population of higher-functioning mitochondria in mother cells by anchorage to the mother cell tip. Defects in either tether compromise asymmetric inheritance of mitochondria and, in turn, lifespan.

Several outstanding questions remain. Mfb1 is the only known protein in yeast that localizes to the mother tip throughout the cell cycle and to the bud tip in telophase. It may therefore interact with thus far uncharacterized polarity cues in yeast. Moreover, the differential localization of old and new mitochondria in human mammary stem-like cells prompts the speculation that tethers between mitochondria and ER in the nuclear envelope and/or PM may contribute to segregation of mitochondria during asymmetric cell division in this stem cell model, and potentially other mammalian cells. Future studies will explore these questions, identify the mechanisms for Mmr1- and Mfb1-mediated mitochondrial tethering, reveal tethers for mitochondria-PM interactions in mammalian cells, and determine whether these tethers contribute to asymmetric inheritance of mitochondria, lifespan control and/or cellular fitness by localizing the most functional mitochondria to their sites of action.

## Author contributions

Each of the authors contributed to writing and editing the mini-review. WP prepared the figures in addition to writing the manuscript.

### Conflict of interest statement

The authors declare that the research was conducted in the absence of any commercial or financial relationships that could be construed as a potential conflict of interest.
